# Caffeine activates HOG-signalling and inhibits pseudohyphal growth in *Saccharomyces cerevisiae*

**DOI:** 10.1186/s13104-023-06312-3

**Published:** 2023-04-14

**Authors:** Tarek Elhasi, Anders Blomberg

**Affiliations:** Lundberg Laboratory, Department of Chemistry and Molecular Biology, University of Gothenburg, Medicinaregatan 9C, Box 462, 405 30 Gothenburg, Sweden

**Keywords:** *Saccharomyces cerevisiae*, Caffeine, Hog1, Phosphorylation, Filamentous/pseudohyphal growth

## Abstract

**Objective:**

Caffeine has a wide range of effects in humans and other organisms. Caffeine activates p38 MAPK, the human homolog to the Hog1 protein that orchestrates the high-osmolarity glycerol (HOG) response to osmotic stress in the yeast *Saccharomyces cerevisiae*. Caffeine has also been used as an inducer of cell-wall stress in yeast via its activation of the Pkc1-mediated cell wall integrity (CWI) pathway. In this study, using immunodetection of phosphorylated Hog1, microscopy to score nuclear localisation of GFP-tagged Hog1 and a pseudohyphal growth assays, the effect of caffeine on the HOG-pathway and filamentous growth in yeast was studied.

**Results:**

It was found that caffeine causes rapid, strong and transient Hog1 dual phosphorylation with statistically significant increases at 20, 30 and 40 mM caffeine. In response to caffeine treatment Hog1 was also rapidly localized to the nucleus, supporting the caffeine-induced phosphorylation and activation of Hog1. We also found that caffeine inhibited the pseudohyphal/filamentous growth in diploid cells, but had no effect on invasive growth in haploids. Our data thus highlights that the HOG signalling pathway is activated by caffeine, which has implications for interpreting caffeine responses in yeast and fungi.

**Supplementary Information:**

The online version contains supplementary material available at 10.1186/s13104-023-06312-3.

## Introduction

Caffeine is a purine analogue of the methylxanthine class that naturally exists as a secondary metabolite in many plants, like in species of the genus *Coffea* from which the popular coffee drink is made. In plants, caffeine has a protective function acting as a pesticide paralyzing and killing insects feeding on the plant [1]. Caffeine has both positive and negative effects on human health, and authoritative agencies therefore continue to identify common healthmetrics and recommended safe intake levels [2]. Caffeine modulates a variety of neurotransmitter systems e.g. reflected in preventing sleepiness and change in the electroencephalographic event-related potentials [3]. The action of caffeine in humans involve three fundamental mechanisms: intracellular mobilization of calcium, inhibition of phosphodiesterases, and antagonism of adenosine receptors [4].

The yeast *Saccharomyces cerevisiae* is a unicellular eukaryote that is a versatile and robust model organism to study fundamental factors that determine eukaryotic cell biology. This yeast is highly responsive to caffeine, with reported effects on cell growth, morphology, DNA repair, intracellular calcium homeostasis, as well as cell cycle progression [5]. It has been proposed that caffeine is transported into the cell by the purine-cytosine permease Fcy2 [6–8] while efflux goes via the transporters Snq2 and Pdr5 [9]. A central effect of caffeine in *S. cerevisiae* is that it activates the Pkc1-mediated cell wall integrity (CWI) pathway via phosphorylation of the down-stream protein kinase Slt2/Mpk1 [10]. Further support for that caffeine is a cell wall perturbing agent in yeast, much in line with congo red and calcofluor-white, is indicated by mutants lacking either surface sensors or components of the CWI signalling pathway are sensitive to caffeine [11].

The response to osmo-stress in yeast is a complex process that is mainly orchestrated by the high osmolarity glycerol (HOG)-pathway, where the protein kinase Hog1 is the final kinase. Hog1 has the human orthologue p38 MAPK (Mitogen Activated Protein Kinase) that can functionally replace Hog1 in yeast [12]. The HOG pathway has also been implicated in the regulation of the yeast cell wall; overactivation of the HOG pathway, by either overexpression of Pbs2 or disruption of the gene encoding the phosphatase Ptc1 (that opposes the action of Pbs2), results in a reduction in the level of 1,6-β-glucan in the cell wall [13]. In human, caffeine activates p38 MAPK via phosphorylation [14]. In yeast, the orthologue of human p38, Hog1, seems to play a role in the response to caffeine, since the deletion of the *HOG1* gene decreased the resistance to caffeine in both *C. albicans* [15] and *S. cerevisiae* [16]. Despite the homology between p38 and Hog1, the effect of caffeine on Hog1 activation in yeast has not yet been studied.

Under starvation to some nutrients, *S. cerevisiae* cells of the sigma strain shift the growth from single cells to form filaments [17]. Thus, it is a dimorphic yeast where the multicellular filamentous state (i.e. pseudohypha) presumably is a foraging mechanism under conditions of nutritional stress [18]. This filamentous growth differs between diploid and haploid cells. In diploid cells it occurs mainly under nitrogen starvation and the filaments extends from the colony, while in haploid cells filamentation occurs under carbon starvation and when cells invade agar. Both forms of growth are pseudohyphal and in both cases a filamentous growth MAPK (fMAPK) cascade is central, starting with activation of Cdc42, followed by regulation of the Ste20, Ste11, Ste7 and Kss1 proteins and their downstream transcription factors Ste12 and Tec1 [19].

Despite the use of caffeine as a genotoxic agent in yeast for many decades, the molecular mechanisms underlying its adverse effects on cell physiology is not fully elucidated. Both the HOG pathway and the filamentous growth pathway are activated by different stimuli that affect the cell wall [20]. Since caffeine is a cell wall stress agent in yeast, we expected that caffeine would act on the HOG- and the filamentous growth pathways. In this study we report that caffeine caused Hog1 phosphorylation that leads to nuclear localisation and inhibited pseudohyphal growth in yeast. Our data thus indicate that caffeine not only affects the CWI pathway, but also impacts on the activity of two other central signalling pathways—HOG and fMAPK—in yeast.

## Main text

### Methods

#### Strains and growth conditions

Standard laboratory strains of *Saccharomyces cerevisiae* were used in all experiments. In studies on Hog1 phosphorylation the strain used was the diploid BY4743 (*MATa/α his3Δ1/his3Δ1 leu2Δ0/leu2Δ0 LYS2/lys2Δ0 met15Δ0/MET15 ura3Δ0/ura3Δ0*). The strain for studies of localization of GFP-tagged Hog1 comes from the genome-wide GFP-collection created in the haploid strain ATCC 201388 (*MATa his3D1 leu2D0 met15D0 ura3D0*) [21]. The strain used in studying pseudohyphal growth was the diploid Σ1278b (*MATa/α ura352/ura352*), while for invasive growth the haploid Σ1278b (*MATa leu2::hisG trp1::hisG his3::hisG ura352*) was used.

All cultures were grown at 30 °C. Studies on caffeine-dependent Hog1 phosphorylation and Hog1-GFP localization were conducted in yeast synthetic YNB media: 0.17% yeast nitrogen base, 0.5% (142 mM) ammonium sulfate, 2% glucose, pH 5.8, buffered with 18.4 mM succinic acid and 0.15 M sodium hydroxide, supplemented with amino acids by addition of 0.4 g/l of CSM complete (#DCS0019; Formedium). Caffeine (Sigma C8960) was added to indicated concentrations (range 3–40 mM).

For studying the effect of caffeine on pseudohyphal formation, we used Synthetic Low Ammonia Dextrose medium [SLAD, 2% glucose, 50 μM ammonium sulfate, 0.17% YNB without amino acids or ammonium sulfate, supplemented with amino acids by addition of 0.4 g/l of CSM complete [#DCS0019; Formedium], 1% agar) [22]. In this method, a 300 mM stock solution of caffeine was spread out on the SLAD agar plates and left to dry for 3 h, to obtain the final concentrations 5 mM (0.25 ml stock, 0.25 ml water) or 10 mM (0.5 ml stock) caffeine. For the negative control 0.5 ml water was spread out on the SLAD plates. We also tested the impact from 100 µg/ml calcofluor white (Sigma F3543) on pseudohyphal growth. Cells were streaked out on the plates and incubated at 30 °C and analysed after 1 and 2 days. For invasive growth analysis, cells were streaked on YPD plates (1% yeast extract, 2% bacterial peptone, 2% glucose, 1% agar) containing two different concentrations of caffeine (3 or 10 mM) for 1 day, after which colonies were washed and scraped by hand to reveal if the cells have invaded the agar (could then not fully be scraped off).

#### Western blot analysis

A stationary-phase overnight culture was inoculated to OD_600_ 0.1 in 5 ml synthetic YNB medium (see above) without caffeine in 15 ml Falcon tubes and incubated with shaking at 30 °C. To exponentially growing cells, OD_600_ 1–1.5, either caffeine-stock or water were added and cultures were incubated in different concentrations of caffeine (10, 20, 30 or 40 mM) for 5, 20 and 60 min. Western blot analysis was performed according to published procedure [23] with the following modifications: 20 µg of protein extract was loaded into 10% Criterion TX precast gels (Bio-Rad) and blotted onto nitrocellulose membrane (Bio-Rad), and dually phosphorylated Hog1 was detected using anti-phospho p38 (Thr180/Tyr182) monoclonal antibodies from rabbit against (Cell Signalling Technology) with 1:1000 dilution. Total Hog1 protein was detected using anti-Hog1 yC-20 from goat (Santa Cruz Biotechnology) with 1:8000 dilution. Bands were quantified using the image analysis program ImageJ, with internal normalisation of the Hog1 phosphorylation to the intensity in the corresponding non-phosphorylated Hog1 in that sample.

#### Microscopy

In order to visualize the localization of Hog1 under caffeine stress, the cultures were cultivated in the same way as indicated above for Western blot analysis, with the difference that only 2 ml medium was used in the final experimental tubes. Caffeine was then added to a final concentration of 10 mM, and the cultures incubated for 5 min at room temperature, where after they were visualized by epiflourescence microscopy. The epifluorescent microscope was equipped with PLAN APOCHROMAT 100×/1.4 Oil DIC lens. The experiment was independently repeated twice (n = 2) and the numbers presented on nuclear localisation was based on counting ≈ 95 cells in each sample.

### Results

#### Caffeine provoked phosphorylation of Hog1

**Fig. 1:**
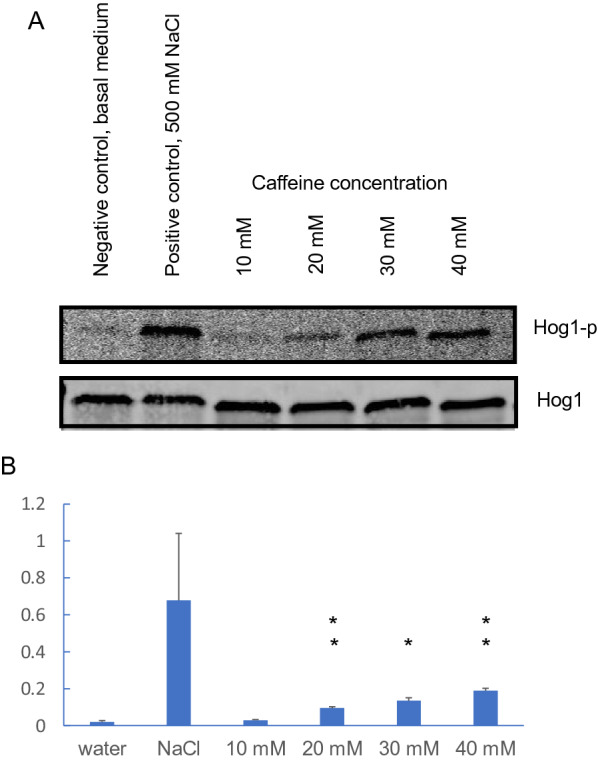
Hog1 is rapidly phosphorylated upon caffeine treatment. **A** Western blot analysis showing Hog1 dual-phosphorylation in response to caffeine. Exponentially growing wild type cells (BY4743) in synthetic medium without caffeine were treated with media with different concentrations of caffeine (10, 20, 30, 40 mM) for 5 min. Negative control was basal growth medium without any caffeine addition, and positive control is treatment with medium containing 500 mM NaCl for 5 min. The upper blot shows the Western signal with the dually phosphorylated Hog1 detected using anti-phospho p38 (Thr180/Tyr182) monoclonal antibodies. The lower blot shows the loading control of total Hog1 detected by Hog1 specific antibodies (non-phosphoryalted form). A typical result is shown from two independent replicates. See Additional file 2: Figure S2 for whole gel displays. **B** Quantification of the two independent analyses, where the intensity of the Hog1-phosphorylated bands for each experiment has been normalised to the Hog1 non-phosphorylated bands in that experiment. Error bars indicate standard deviation. Student t-test was performed and stars indicate statistically significant differences compared to the control/water (one star: p < 0.05; two stars: p < 0.01)

In order to examine if caffeine can cause Hog1 phosphorylation, different concentrations of caffeine were applied to exponentially growing yeast cells and phosphorylated Hog1 was detected by Western analysis. The lowest concentration of caffeine tested, 10 mM, caused a reduction in growth rate with roughly 125% [generation time: control 2.1 ± 0.2 h (± standard deviation), and with caffeine 4.8 ± 0.7 h; n = 2; 5 technical replicates for each; for complete growth curves see Additional file 1: Figure S1]. A weak band of dual-phosphorylated Hog1 was observed in basal control medium and at 10 mM of caffeine, however, the band intensity steadily increased in a caffeine-dependent manner by increasing the caffeine concentration to 20, 30, and 40 mM (Fig. 1A; Additional file 2: Figure S2) with statistically significant increases at these higher caffeine concentrations (Fig. 1B). The Hog1 phosphorylation was analysed during different times of exposure (5, 20 and 40 min) and was found to be strongest at 5 min (data not shown). At 40 mM caffeine the phosphorylation of Hog1 was ninefold higher than in the control without caffeine. However, the signal at 40 mM was roughly 30% of the positive control 500 mM NaCl indicating that at these concentrations of external stressors NaCl was a stronger inducer of Hog1 phosphorylation than caffeine. The same amount of total Hog1 protein was detected under all test-conditions. We conclude that caffeine treatment led to a rapid, strong and transient phosphorylation of Hog1.

#### Hog1 was directed to the nucleus under caffeine stress

**Fig. 2:**
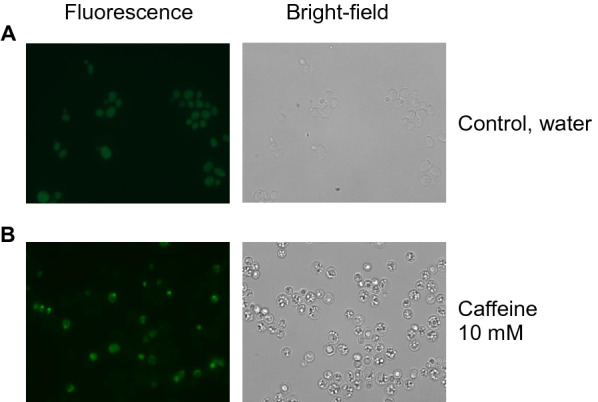
Localization of GFP-tagged Hog1 in response to caffeine. Left: Fluorescent image, Right: bright-field image (**A**) in water, control (exponentially growing cells) (**B**) in 10 mM caffeine for 5 min. Under caffeine stress, Hog1 was transferred to the nucleus. A typical result is shown from two independent replicates. Microscopic magnification for all images is 400×

Upon phosphorylation in response to osmotic stress, Hog1 is directed to the nucleus [12]. In order to know where the phosphorylated Hog1 will be directed in response to caffeine stress, GFP-tagged Hog1 cells were observed under the epiflourescence microscope, with and without caffeine. During exponential growth in basal medium Hog1-GFP is evenly localized throughout the whole cell (no accumulation in any of the cell nuclei), while Hog1-GFP rapidly accumulated (within 5 min) in the nucleus in response to 10 mM caffeine in 23 ± 8% of the cells (n = 2) (Fig. 2).

#### Caffeine inhibited pseudohyphal growth in diploids but not invasive growth in haploids

In order to know if caffeine can affect the pseudohyphal growth in diploid yeast cells, caffeine was applied to diploid Σ1278 cells in SLAD media; a low nitrogen medium that induces pseudohyphal growth [22]. The strain Σ1278 is the preferred genetic background for studies of filamentous growth since it easily undergoes the morphological transition from single cell to pseudohypha [19].

**Fig. 3:**
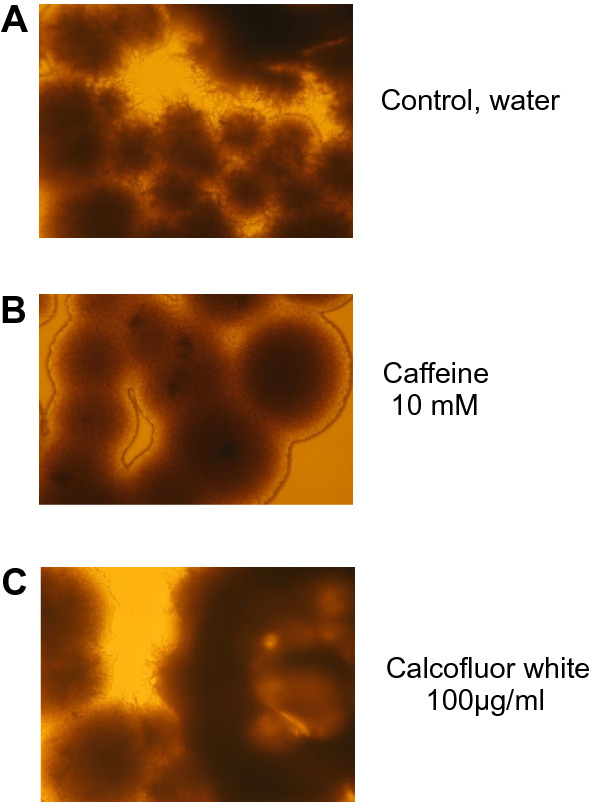
Pseudohyphal growth in diploid cells was inhibited by caffeine but not by calcofluor white. The diploid strain Σ1278 was in cultured on solid SLAD media at 30C for 2 days. **A** Control (water), **B** caffeine (10 mM) and **C** calcofluor white (100 µg/ml). A typical result is shown from two independent replicates, each containing two technical replicates. Microscopic magnification for all images is 100×

We observed that 10 mM caffeine inhibited pseudohyphal extensions in the diploid strain and the colony edges were smooth (Fig. 3). This effect was observed even at 3 mM caffeine (Additional file 3: Figure S3). The growth of the colonies continued even in the presence of caffeine, indicating that caffeine had no adverse effects on cell division (data not shown). Calcofluor white is a fluorescent agent that perturbs the cell wall by strongly binding to cell wall chitin and in that way interfering with chitin synthesis [24]. We used calcoflour white as a control for general cell wall perturbation and found no effect on pseudohyphal growth. Thus, the caffeine inhibition of pseudohypha appears not to be the effect of general changes in the cell wall. In addition, in contrast to the impact on pseudohyphal growth of diploid cells, caffeine did not inhibit invasive growth in haploid cells, not even at 30 mM (Additional file 4: Figure S4). Thus, the morphogenic effect of caffeine appeared to be specific for inhibition of pseudohyphal growth in diploids.

### Discussion

We here report on two novel yeast phenomena in response to treatment with the purine analogue caffeine. Firstly, we show that caffeine causes Hog1 phosphorylation in a dose-dependent manner. This was directly observed since phosphorylated Hog1 was detected by Western blot with an antibody specific for dual phosphorylated Hog1 (Thr180/Tyr182), but also indirectly supported by the caffeine-induced translocation of the GFP-tagged Hog1 to the nucleus. Several earlier studies have shown that phosphorylation of Hog1 is a prerequisite for its nuclear localisation [12]. Secondly, we also report that caffeine inhibits pseudohyphal growth in diploid cells even at low concentrations.

We have not investigated the relation between Hog1 and pseudohyphal development, however, a potential mechanistic connection between these two caffeine-responding phenomena is supported by the earlier report showing that *hog1∆/hog1∆* diploids exhibit hyper-pseudohyphal growth [25]. Further support for an inhibitory role of Hog1 in filament formation comes from a study that showed activated Hog1 to be an inhibitor of pseudohyphal growth [26]. These authors also reported that overexpression of several proteins that promote export of Hog1 from the nucleus, e.g. Nbo2, Ptp2, and Crm1, leads to enhanced pseudohyphal development. Interestingly, in a genome-wide screen of deletion mutants in the sigma-strain background, Hog1 and Hog1-related mutants were identified as promoting hyper-pseudohyphal growth, i.e. *hog1∆, gpp1∆* (glycerol 3-phosphatase), *ssk2∆* (MAP kinase kinase kinase of HOG signaling pathway) and *frt2∆* (ER membrane protein of unknown function that promotes growth in high Na^+^) [27], further stressing the importance of the HOG pathway and its down-stream components in filamentous growth. Enhanced osmolarity activates Hog1 [12]. At this stage, the relation between osmotic pressure (e.g. addition of NaCl or sorbitol) and pseudohyphal growth has not been investigated for *S. cerevisiae*. However, it has been established that osmolarity-equivalent concentrations of these stress-agents produce a reduction of pseudohyphae growth of *Candida lusitaniae* [28] and their influence on pseudohyphal development in *S. cerevisiae* certainly deserves future attention. Thus, our working hypothesis is that caffeine leads to Hog1 phophorylation and activation, and that this Hog1 activation inhibits pseudohyphal development.

There are roughly 570 genes that respond to increased osmolarity in a Hog1-dependent manner, however, only a subset of those (32 genes) appears to be fully Hog1-dependent [29, 30]. Examining the published expression changes upon caffeine addition [5] (https://www.ncbi.nlm.nih.gov/sites/GDSbrowser?acc=GDS2914) we found that fully Hog1-dependent genes like *STL1, TKL2, GRE2, DAK1* and *PGM2* do not respond to caffeine. Thus, the here reported Hog1 activation by caffeine is not sufficient for their induction, indicating that other mechanisms need to be in play to combat/counteract the Hog1 activation under caffeine treatment. Global gene expression studies have shown that the TOR-complex is a likely target of caffeine, since the expression changes from treatment with either caffeine or rapamycine (a known inhibitor of TOR) exhibit rather similar expression responses [5, 31]. The expression profiles for the two drugs are rather similar both for repression (e.g. ribosomal protein genes like *RPL30*) as well as induction (e.g. genes involved in allantoin degradation like *DAL2* and *DAL5*) [31]. However, several unique expression differences were observed for each drug indicating also drug-specific targets. Further support that caffeine and rapamycin have somewhat different targets comes from the fact that some point-mutations in Tor1 that leads to rapamycin resistance, are still hypersensitive to caffeine [31].

How caffeine activates Hog1 is not known, however, it has been reported that Hog1 can be activated by several external factors that affect cell wall dynamics (reviewed in [20]). However, earlier report has shown that calcofluor white treatment, which impacts on the cell wall, failed to activate the HOG pathway [32]. The inner layer of the cell wall is mainly composed of glucan and chitin, which are together mainly responsible for the strength and elasticity of the cell wall [33]. A study by Kuranda et al. found that during prolonged incubation with caffeine the content of glucan and chitin in the cell wall is altered [5]. These authors also found that caffeine induces Slt2/Mpk1 phosphorylation, the final protein kinase in the cell wall integrity pathway and concluded that this phosphorylation is a result of cell wall damage by caffeine, because it was prevented in the presence of osmotic support [10].

Even if caffeine clearly inhibited pseudohyphal growth in diploids it did not inhibit invasive growth in haploids. Why did caffeine have a different effect in diploid vs haploid cells? There are several indications that invasive growth in haploids and pseudohyphal growth in diploids have many signalling components in common, encompassing the MAPK pathway with Kss1, the Snf1 pathway, the TOR pathway and the RAS/PKA pathway [19]. The constitutively activated Ras2-val19 mutation enhances pseudohyphal growth in diploids as well as invasive growth in haploids [34]. However, there are also distinct mechanistic differences: (i) a *ras2* mutation prevents haploid invasive growth while a *ras2/ras2* homozygous mutation allows pseudohyphal growth in diploids; (ii) a knockout of the cyclin *CLN1* does not block invasive growth in haploids while a *cln1/cln1* diploid strain is unable to induce pseudohyphae upon stimulation; and (iii) several RIM mutants (regulator of Ime2) inhibits invasive growth in haploids while in homozygous diploids there is no defect in pseudohyphal growth. Furthermore, the deletion of components of the amino acid sensor system, Ptr3 or Ssy1, causes constitutive haploid invasive growth but not diploid pseudohyphal differentiation [35]. Thus, it is possible that caffeine interferes with one of the pathways that regulate filamentous growth in diploid while not affecting the pathways important for pseudohyphal differentiation, i.e. invasive growth, in haploids.

## Limitations

Different strain backgrounds were used in the different functional analyses, however, this is a rather common procedure in the yeast field. Filamentous growth was studied in the sigma-strain, which is the classical strain that shows a clear filamentous response upon nutrient starvation. For Hog1 localisation studies the strain was ATCC 201388, which is the background in which the GFP-tagged protein-library was made [21], while for Western analysis the commonly used laboratory BY strain was used. However, all strains are derived from the ancestor strain S288C, which makes them closely related and they should therefore not deviate too much in their responses.

## Supplementary Information

**Additional file 1: Figure S1.** Growth of yeast cells (strain BY4743) with (+ caf; caffeine concentration 10 mM) and without (-caf) caffeine. Growth was followed at 30 °C by microcultivation in 350 μl synthetic YNB medium in a Bioscreen C (automated shaking and optical density readings), with optical density (OD) measurements every 20 min. Inoculation to cultures from a small number of cells taken from an overnight colony on synthetic YNB agar plates (without caffeine). The growth analysis was performed in two independent replicates (n = 2) with in each case 5 technical replicates. Typical growth curves are displayed.

**Additional file 2: Figure S2.** Western blot analysis showing Hog1 dual-phosphorylation in response to caffeine. Exponentially growing wild type cells (BY4743) in synthetic medium without caffeine were treated with media with different concentrations of caffeine (10, 20, 30, 40 mM) for 5 min. Negative control was basal growth medium without any caffeine addition, and positive control is treatment with medium containing 500 mM NaCl for 5 min. A) Hog1-p detected with dualphosphorylated-specific antibody. B) Loading control of total Hog1 detected by Hog1 specific antibodies (non-phosphorylated form). Molecular weight standard to the left-most lane in both cases. A typical result is shown from two independent replicates.

**Additional file 3: Figure S3.** Pseudohyphal growth in diploid cells was inhibited by caffeine. The diploid strain Σ1278 was cultured on solid SLAD media at 30 °C for 2 days. Upper panel control (water), lower panels: different concentration of caffeine (3, 5, 7, 10 mM). The edge of the colony (indicated by arrows) of the control shows filamentous extensions, while the edges of colonies grown in the presence of different concentrations of caffeine appeared smooth and even. A typical result is shown from two independent replicates, each containing two technical replicates for the control and 10 mM caffeine, while 3, 5 and 7 mM caffeine is a typical result from one run with two technical replicates. The original microscopic magnification for all images is 100×. However, in this supplementary figure selected portions of the images have been further enhanced (zoomed-in) to emphasis the colony edge-effects, resulting in another 10× magnification compared to the images presented in Figure 3 in the main text.

**Additional file 4: Figure S4.** Invasive growth under caffeine stress. Cells of the haploid Σ1278 strain were grown on YPD plates containing different concentrations of caffeine (3 and 10 mM). A typical result is shown from two independent replicates, each containing two technical replicates.

## Data Availability

The datasets used and/or analysed during the current study are available from the corresponding author on reasonable request.

## Abbreviations

HOG: High osmolarity glycerol
MAPK: Mitogen activated protein kinase
SLAD: Synthetic low ammonia dextrose medium
RAS/PKA: The Ras2p/cAMP-dependent protein kinase A
Snf1: The AMP-activated protein kinase (AMPK) pathway
TOR: The target of rapamycin
