# Supplementary material for: Caffeine activates HOG-signalling and inhibits pseudohyphal growth in Saccharomyces cerevisiae
Source: BMC Res Notes. 2023 Apr 14;16:52. doi: 10.1186/s13104-023-06312-3 (PMC10105414; doi:10.1186/s13104-023-06312-3)
Supplement: Supplementary file 1 — Additional file 1: Figure S1. Growth of yeast cells (strain BY4743) with (+ caf; caffeine concentration 10 mM) and without (-caf) caffeine. Growth was followed at 30 °C by microcultivation in 350 μl synthetic YNB medium in a Bioscreen C (automated shaking and optical density readings), with optical density (OD) measurements every 20 min. Inoculation to cultures from a small number of cells taken from an overnight colony on synthetic YNB agar plates (without caffeine). The growth analysis was performed in two independent replicates (n = 2) with in each case 5 technical replicates. Typical growth curves are displayed. [file 13104_2023_6312_MOESM1_ESM.pdf]

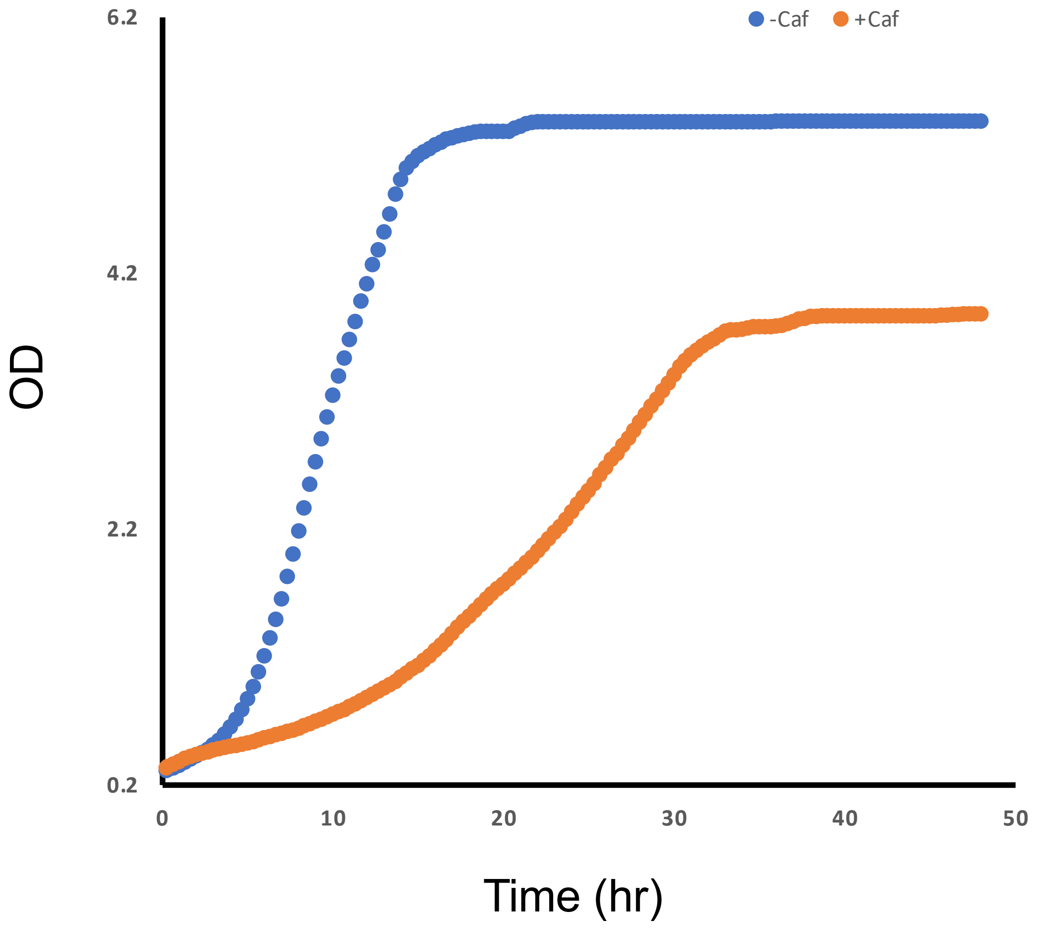

Figure S1. Growth of yeast cells (strain BY4743) with (+caf; caffeine concentration 10 mM) and without (-caf) caffeine. Growth was followed at 30°C by microcultivation in 350µl synthetic YNB medium in a Bioscreen C (automated shaking and optical density readings), with optical density (OD) measurements every 20 minutes. Inoculation to cultures from a small number of cells taken from an overnight colony on synthetic YNB agar plates (without caffeine). The growth analysis was performed in two independent replicates (n=2) with in each case 5 technical replicates. Typical growth curves are displayed.
