# Supplementary material for: Caffeine activates HOG-signalling and inhibits pseudohyphal growth in Saccharomyces cerevisiae
Source: BMC Res Notes. 2023 Apr 14;16:52. doi: 10.1186/s13104-023-06312-3 (PMC10105414; doi:10.1186/s13104-023-06312-3)
Supplement: Supplementary file 2 — Additional file 2: Figure S2. Western blot analysis showing Hog1 dual-phosphorylation in response to caffeine. Exponentially growing wild type cells (BY4743) in synthetic medium without caffeine were treated with media with different concentrations of caffeine (10, 20, 30, 40 mM) for 5 min. Negative control was basal growth medium without any caffeine addition, and positive control is treatment with medium containing 500 mM NaCl for 5 min. A) Hog1-p detected with dualphosphorylated-specific antibody. B) Loading control of total Hog1 detected by Hog1 specific antibodies (non-phosphorylated form). Molecular weight standard to the left-most lane in both cases. A typical result is shown from two independent replicates. [file 13104_2023_6312_MOESM2_ESM.pdf]

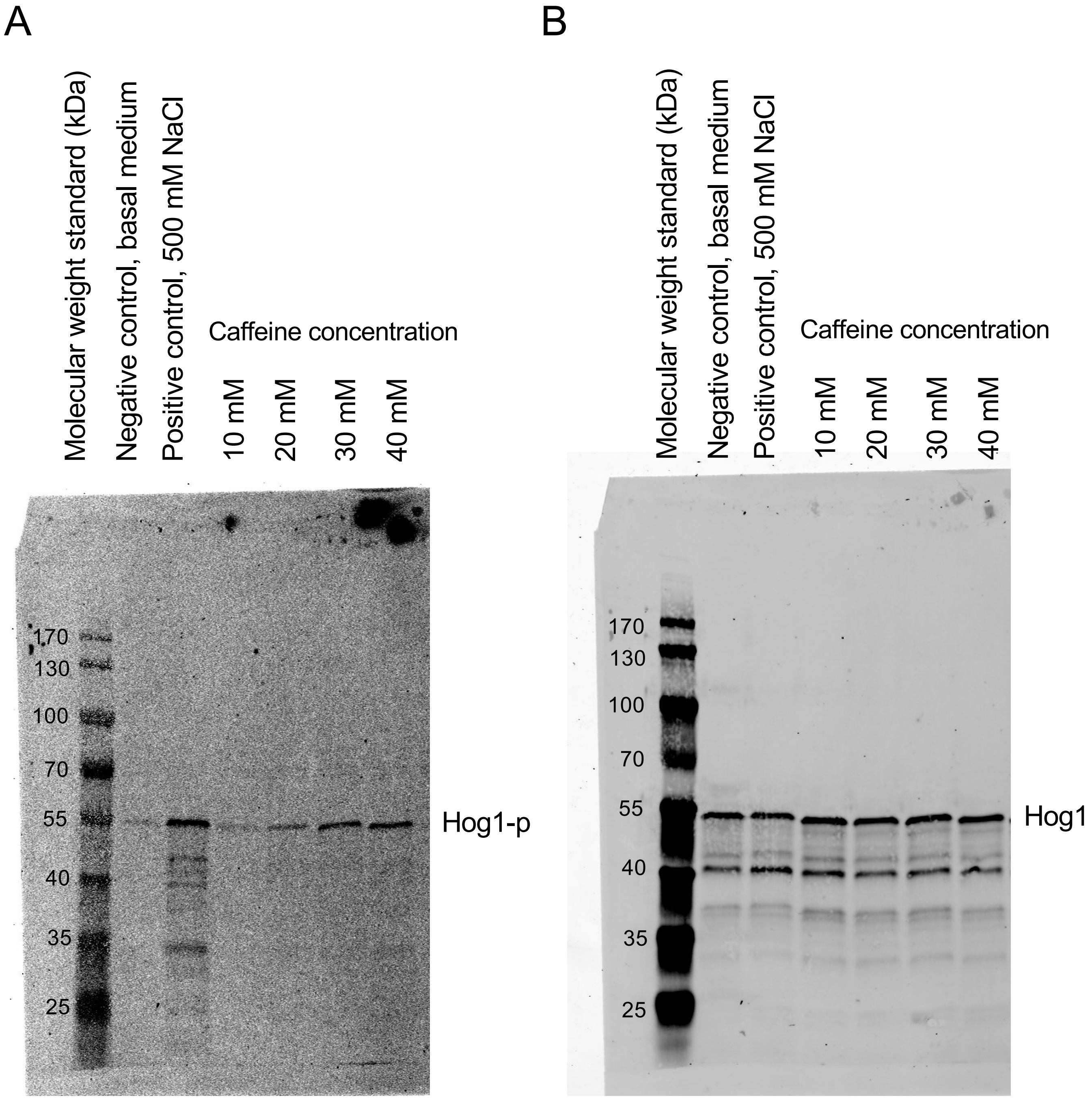

Figure S2. Western blot analysis showing Hog1 dual-phosphorylation in response to caffeine. Exponentially growing wild type cells (BY4743) in synthetic medium without caffeine were treated with media with different concentrations of caffeine (10, 20, 30, 40 mM) for 5 min. Negative control was basal growth medium without any caffeine addition, and positive control is treatment with medium containing 500 mM NaCl for 5 min. A) Hog1-p detected with dual-phosphorylated-specific antibody. B) Loading control of total Hog1 detected by Hog1 specific antibodies (non-phosphorylated form). Molecular weight standard to the left-most lane in both cases.
A typical result is shown from two independent replicates.
