# Supplementary material for: Caffeine activates HOG-signalling and inhibits pseudohyphal growth in Saccharomyces cerevisiae
Source: BMC Res Notes. 2023 Apr 14;16:52. doi: 10.1186/s13104-023-06312-3 (PMC10105414; doi:10.1186/s13104-023-06312-3)
Supplement: Supplementary file 3 — Additional file 3: Figure S3. Pseudohyphal growth in diploid cells was inhibited by caffeine. The diploid strain Σ1278 was cultured on solid SLAD media at 30 °C for 2 days. Upper panel control (water), lower panels: different concentration of caffeine (3, 5, 7, 10 mM). The edge of the colony (indicated by arrows) of the control shows filamentous extensions, while the edges of colonies grown in the presence of different concentrations of caffeine appeared smooth and even. A typical result is shown from two independent replicates, each containing two technical replicates for the control and 10 mM caffeine, while 3, 5 and 7 mM caffeine is a typical result from one run with two technical replicates. The original microscopic magnification for all images is 100×. However, in this supplementary figure selected portions of the images have been further enhanced (zoomed-in) to emphasis the colony edge-effects, resulting in another 10× magnification compared to the images presented in Figure 3 in the main text. [file 13104_2023_6312_MOESM3_ESM.pdf]

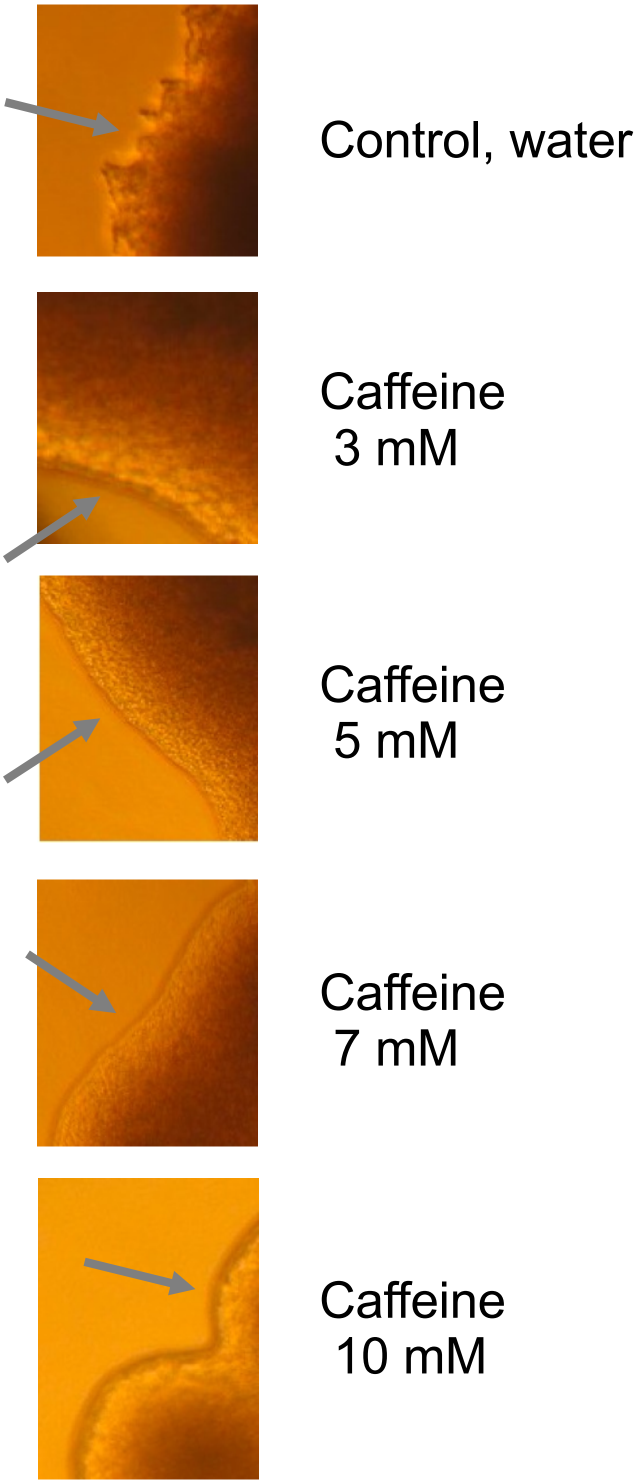

Figure S3. Pseudohyphal growth in diploid cells was inhibited by caffeine. The diploid strain Σ1278 was cultured on solid SLAD media at 30°C for 2 days. Upper panel control (water), lower panels: different concentration of caffeine (3, 5, 7, 10 mM). The edge of the colony (indicated by arrows) of the control shows filamentous extensions, while the edges of colonies grown in the presence of different concentrations of caffeine appeared smooth and even. A typical result is shown from two independent replicates, each containing two technical replicates for the control and 10 mM caffeine, while 3, 5 and 7 mM caffeine is a typical result from one run with two technical replicates. The original microscopic magnification for all images is 100x. However, in this supplementary figure selected portions of the images have been further enhanced (zoomed-in) to emphasis the colony edge-effects, resulting in another 10x magnification compared to the images presented in Figure 3 in the main text.
