# Supplementary material for: Caffeine activates HOG-signalling and inhibits pseudohyphal growth in Saccharomyces cerevisiae
Source: BMC Res Notes. 2023 Apr 14;16:52. doi: 10.1186/s13104-023-06312-3 (PMC10105414; doi:10.1186/s13104-023-06312-3)
Supplement: Supplementary file 4 — Additional file 4: Figure S4. Invasive growth under caffeine stress. Cells of the haploid Σ1278 strain were grown on YPD plates containing different concentrations of caffeine (3 and 10 mM). A typical result is shown from two independent replicates, each containing two technical replicates. [file 13104_2023_6312_MOESM4_ESM.pdf]

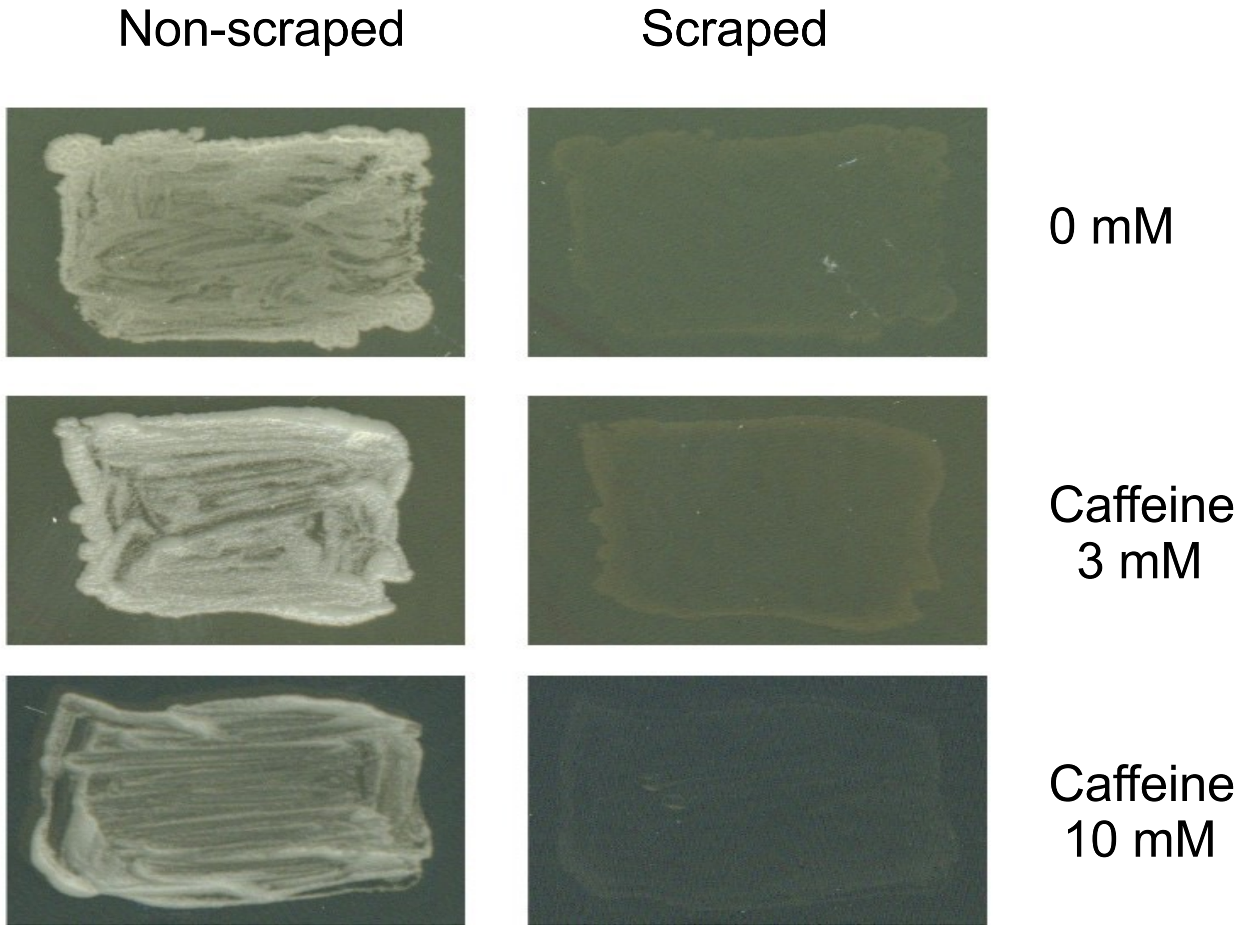

Figure S4. Invasive growth under caffeine stress. Cells of the haploid Σ1278 strain were grown on YPD plates containing different concentrations of caffeine (3 and 10 mM). A typical result is shown from two independent replicates, each containing two technical replicates.
